# Ribosome biogenesis and degradation regulate translational capacity during muscle disuse and reloading

**DOI:** 10.1002/jcsm.12636

**Published:** 2020-11-24

**Authors:** Vandré C. Figueiredo, Randall F. D'Souza, Douglas W. Van Pelt, Marcus M. Lawrence, Nina Zeng, James F. Markworth, Sally D. Poppitt, Benjamin F. Miller, Cameron J. Mitchell, John J. McCarthy, Esther E. Dupont‐Versteegden, David Cameron‐Smith

**Affiliations:** ^1^ Liggins Institute The University of Auckland Auckland New Zealand; ^2^ Department of Physical Therapy, College of Health Sciences University of Kentucky KY USA; ^3^ Center of Muscle Biology University of Kentucky KY USA; ^4^ College of Medicine University of Kentucky KY USA; ^5^ Aging and Metabolism Research Program Oklahoma Medical Research Foundation (OMRF) Oklahoma City OK USA; ^6^ School of Kinesiology University of British Columbia Vancouver Canada; ^7^ School of Biological Sciences The University of Auckland Auckland New Zealand; ^8^ Human Potential Translational Research Programme, Yong Loo Lin School of Medicine National University of Singapore Singapore; ^9^ Singapore Institute for Clinical Sciences Agency for Science, Technology and Research Singapore

**Keywords:** Ribosomal RNA, Ribophagy, Atrophy, Regrowth, Resistance training

## Abstract

**Background:**

Translational capacity (i.e. ribosomal mass) is a key determinant of protein synthesis and has been associated with skeletal muscle hypertrophy. The role of translational capacity in muscle atrophy and regrowth from disuse is largely unknown. Therefore, we investigated the effect of muscle disuse and reloading on translational capacity in middle‐aged men (Study 1) and in rats (Study 2).

**Methods:**

In Study 1, 28 male participants (age 50.03 ± 3.54 years) underwent 2 weeks of knee immobilization followed by 2 weeks of ambulatory recovery and a further 2 weeks of resistance training. Muscle biopsies were obtained for measurement of total RNA and pre‐ribosomal (r)RNA expression, and *vastus lateralis* cross‐sectional area (CSA) was determined via peripheral quantitative computed tomography. In Study 2, male rats underwent hindlimb suspension (HS) for either 24 h (HS 24 h, *n* = 4) or 7 days (HS 7d, *n* = 5), HS for 7 days followed by 7 days of reloading (Rel, *n* = 5) or remained as ambulatory weight bearing (WB, *n* = 5) controls. Rats received deuterium oxide throughout the study to determine RNA synthesis and degradation, and mTORC1 signalling pathway was assessed.

**Results:**

Two weeks of immobilization reduced total RNA concentration (20%) and CSA (4%) in men (both *P* ≤ 0.05). Ambulatory recovery restored total RNA concentration to baseline levels and partially restored muscle CSA. Total RNA concentration and 47S pre‐rRNA expression increased above basal levels after resistance training (*P* ≤ 0.05). In rats, RNA synthesis was 30% lower while degradation was ~400% higher in HS 7d in soleus and plantaris muscles compared with WB (*P* ≤ 0.05). mTORC1 signalling was lower in HS compared with WB as was 47S pre‐rRNA (*P* ≤ 0.05). With reloading, the aforementioned parameters were restored to WB levels while RNA degradation was suppressed (*P* ≤ 0.05).

**Conclusions:**

Changes in RNA concentration following muscle disuse and reloading were associated with changes in ribosome biogenesis and degradation, indicating that both processes are important determinants of translational capacity. The pre‐clinical data help explain the reduced translational capacity after muscle immobilization in humans and demonstrate that ribosome biogenesis and degradation might be valuable therapeutic targets to maintain muscle mass during disuse.

## Introduction

Muscle disuse, such as occurs during hospitalization, bed rest, or immobilization, causes a rapid decline in skeletal muscle mass, strength and function at all ages.^1^, ^2^, ^3^, ^4^, ^5^, ^6^, ^7^, ^8^ Episodes of temporary disuse are particularly relevant in older individuals, as it can accelerate the typical gradual loss of muscle mass associated with aging or sarcopenia,^2^, ^9^ leading to decreased quality of life and heightened risk of comorbidities and mortality.^10^ Loss of muscle mass typically starts at the fifth decade of life,^11^, ^12^ thus middle‐aged adults are a relevant group in which to study the initial sarcopenic events.^1^ Physical inactivity or muscle disuse is therefore significant in the initiation and progression towards sarcopenia.^13^, ^14^ Conversely, muscle reloading (returning to usual weight‐bearing physical activities) and resistance training following disuse promote muscle regrowth.^15^, ^16^ To date, the molecular mechanisms of muscle atrophy induced by disuse and regrowth during reloading are not completely understood, and controversies on what the primary determinants are still exist in the field.^17^, ^18^ Understanding the molecular regulation of muscle loss and regrowth after a period of muscle disuse can help design better therapeutic approaches to target atrophy.

It is well established that within the first days of disuse both post‐prandial and post‐absorptive rates of muscle protein synthesis are depressed and are not fully normalized even after long‐term periods (several days or weeks) of unloading in both animal models and humans.^15^, ^16^, ^19^, ^20^, ^21^ Diminished translational efficiency (translation rate per ribosome) appears to play a role in attenuated post‐prandial muscle protein synthesis in immobilized human limbs,^19^ but there is a paucity of data regarding the effects of disuse on translational capacity (ribosome content). In rodents, however, it has been shown that prolonged muscle disuse reduces translational capacity.^21^, ^22^, ^23^, ^24^ Similarly, the molecular mechanisms underlying muscle regrowth after atrophy are not completely understood.^15^ The mammalian/mechanistic target of rapamycin complex 1 (mTORC1), which controls the initiation of mRNA translation, appears to be highly involved with muscle regrowth following disuse;^25^ however, other mechanisms, such as ribosome biogenesis, may also contribute to muscle regrowth.^15^, ^21^ To date, the effect of unloading and reloading on translational capacity and muscle ribosome biogenesis in humans has not been investigated. The potential role of ribosome biogenesis affecting translational capacity and muscle mass during muscle disuse and reloading could help explain the differences in post‐absorptive and post‐prandial muscle protein synthesis during unloading and reloading.

Ribosome biogenesis is a central mechanism driving increased muscle cell translational capacity and has been associated with skeletal muscle hypertrophy induced by resistance training^26^, ^27^, ^28^ and with muscle wasting.^29^ Ribosome biogenesis is the cellular process of synthesizing new ribosomes via the transcription of the ribosomal DNA (rDNA) by RNA Polymerase I (Pol I) into the 47S pre‐ribosomal RNA (47S pre‐rRNA), which contains three of the four ribosomal RNAs (18S, 28S, and 5.8S rRNAs) required to form a ribosome, in addition to 5S, which is transcribed by Pol III from the 5S rDNA.^30^, ^31^ Along with ribosomal proteins, the four rRNAs compose the small and large subunits of the mature ribosome. The synthesis of 47S pre‐rRNA by Pol I is a rate‐limiting step in ribosome biogenesis,^32^, ^33^, ^34^ and because it is rapidly processed,^35^ 47S pre‐rRNA levels reflect the rate of rRNA synthesis at a particular point in time.^32^ More recently, the incorporation of deuterium into purine ribose of RNA following labelling with deuterium oxide (D_2_O) has been used to determine the rate of total RNA synthesis and degradation over longer periods of time,^36^, ^37^ which is particularly relevant for ribosome turnover. When labelling for periods of days to weeks, this approach largely measures ribosome biogenesis because the 80–85% of RNA is rRNA, and mRNA and tRNA are largely fully turned over by the first day.^36^, ^37^

To investigate the effects of disuse and reloading in skeletal muscle, we assessed the impact of 2 weeks of unilateral lower limb immobilization followed by 2 weeks of ambulatory recovery and a further 2 weeks of resistance training on ribosome biogenesis and muscle translational capacity in middle‐aged men. Because of the potential limitations of acquiring multiple muscle biopsies in humans, we coupled this study with a rodent model of muscle disuse and recovery, to include RNA turnover measurements and to gain temporal resolution and further mechanistic insight. In particular, we were interested in early time points following the onset of hindlimb suspension (HS) and subsequent reloading. Our overarching hypothesis is that habitual muscle loading maintains ribosome biogenesis and thereby translational capacity in humans and rats, and that, therefore, muscle disuse decreases total RNA concentration (i.e. translation capacity) by changing the balance in RNA turnover. It is further hypothesized that ribosomal transcription precedes changes in translational capacity, and reloading restores the balance in RNA turnover.

## Methods

### Study 1

#### Participants

Twenty‐eight healthy middle‐aged men (age: 50 ± 3.54 years old; height: 178 ± 7.07 cm; weight: 88 ± 12.96 kg; body mass index: 28 ± 3.30 kg·m^−2^) who were sedentary to moderately active and overall healthy (non‐diabetic, non‐smoking, and free of chronic muscle disease or injury) volunteered for the study. The data presented in Study 1 are part of a larger project investigating the effects of immobilization and subsequent rehabilitation on muscle mass and health in middle‐aged men (registered with the Australia New Zealand Clinical Trial Registry # ACTRN12615000454572 on 11 May 2015). Detailed methods and results of primary outcomes have been previously reported.^6^ The study was approved by the Northern Health and Disability ethics committee (New Zealand) and was in compliance with the declaration of Helsinki. All participants were informed of the requirements and possible risks of the research before written informed consent was obtained.

#### Experimental design

**FIGURE 1:**
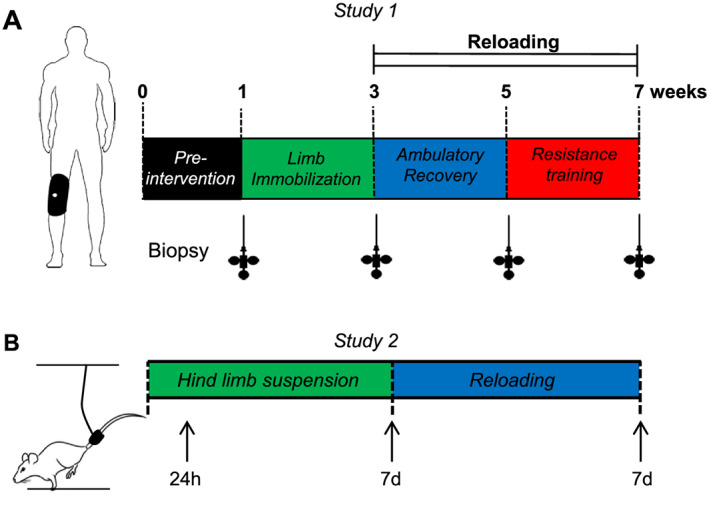
Experimental designs. Timeline of measurements and interventions in Study 1 (A). Muscle biopsies were taken at four occasions: at baseline, following 2 weeks of limb immobilization, 2 weeks of ambulatory recovery, and 2 weeks of resistance training. Timeline of muscle collection in Study 2 (B). Arrows indicate when skeletal muscle was harvested.

The detailed study design has already been described elsewhere,^6^ but an overview of the experimental design is depicted in *Figure* 1A. It is noteworthy that the analysis presented here excluded two participants from the previous report^6^ because skeletal muscle biopsy material for RNA analysis was not available. After screening and familiarization, participants visited the laboratory on four occasions after an overnight fast. During each visit, a biopsy sample was obtained from the *vastus lateralis* muscle, and a peripheral quantitative computed tomography (pQCT) scan (XCT 3000 pQCT scanner, Stratec Medizintechnik GmbH, Pforzheim, Germany) was performed to quantify mid‐thigh muscle cross‐sectional area (CSA). CSA has been presented previously in Mitchell et al.^6^ Herein, muscle CSA data are presented as % change from baseline with the exclusion of the two subjects from whom RNA was not available.

During all phases of the study, participants were provided with standardized dinners and breakfasts. Participants were randomized to consume either 20 g of dairy protein or a carbohydrate‐based placebo control once daily.^6^ Protein supplementation had no significant effect on any measures included in the present study so data were pooled for both groups. Dietary advice was provided for the midday meal in order to ensure participants were in energy balance and consuming 1.1–1.3 g of protein per kilogram of body weight per day throughout the experimental trial.

Participant's habitual activity was measured during a 1‐week diet‐controlled pre‐intervention period using wrist band accelerometers (Fitbit Tracker ™, San Francisco, CA, USA). Following the pre‐intervention period, participants returned to the laboratory, and a muscle biopsy was collected (which was used as baseline biopsy) and a pQCT scan performed. Participants were then subjected to 2‐week unilateral limb immobilization in which one lower limb was fully immobilized at 60° of knee flexion (counter balanced for leg dominance) using a unilateral knee brace (Donjoy IROM; Vista, CA, USA). Velcro straps were used to hold the brace in place, and tape was affixed on top of the straps with an investigators' signature inscribed. Braces were regularly checked at participant's home or at the laboratory to ensure compliance and that they were securely fastened and not causing injury or irritation.

A third muscle biopsy was taken following knee brace removal (post‐immobilization), and pQCT scan was performed. This was followed by 2 weeks of muscle reloading termed ambulatory recovery, in which participants resumed their normal activities. After the ambulatory recovery period, a muscle biopsy was taken, and a third pQCT scan was performed, before participants initiated a resistance training programme for an additional 2 weeks. Resistance exercise sessions were performed thrice weekly. Each participant was individually trained under supervision. The exercise programme consisted of a leg press and knee extension exercises. Participants performed four sets of 10 repetitions at 80% of 1RM (last set until failure) following a warm‐up set performed at 50% of 1RM. Loads were progressed when a participant could complete 4 sets of 10 repetitions with the prescribed load. Two‐minute rest intervals were allowed between sets. Following the resistance training period, a final post‐resistance training biopsy was taken at rest, and pQCT scan was performed. All muscles biopsies were taken under local anaesthesia (1% Xylocaine) using Bergström needle modified from manual suction and were snap‐frozen in liquid nitrogen and stored at −80°C until further analysis. Habitual physical activity was not different between the baseline, ambulatory recovery and resistance training phases as measured by average daily steps (10 337 ± 727; 11 723 ± 611; 11 981 ± 717; respectively).^6^

#### Total RNA extraction and reverse transcription polymerase chain reaction

Total RNA was extracted from ~15 mg of muscle tissue using the AllPrep® DNA/RNA/miRNA Universal Kit (QIAGEN GmbH, Hilden, Germany) following the manufacturer's instructions. RNA concentration was measured using Qubit™ RNA HS Assay Kit. Following complementary DNA (cDNA) synthesis using high‐capacity RNA‐to‐cDNA™ kit (Life Technologies, Carlsbad, CA, USA), ribosomal and messenger RNA (rRNA and mRNA) were measured by reverse transcription polymerase chain reaction (RT‐PCR) on a LightCycler 480 II (Roche Applied Science, Penzberg, Germany) using SYBR Green I Master Mix (Roche Applied Science). Pre‐rRNA was measured using primers designed specifically for pre‐rRNA sequence spanning the 5.8S rRNA and the internal transcribed spacer (ITS) region, designated as ITS‐5.8S rRNA. This primer set was designed by QIAGEN, using RT^2^ Profiler PCR Arrays (QIAGEN). We have previously described these primers ^26^. The geometric mean of three reference genes^38^ was used for normalization. Several reference genes, among recently proposed stable genes,^39^ and genes usually found in the literature as reference genes (*C1orf43*, *CHMP2A*, *EMC7*, *TBP*, *PPIA*, *TCP*, and *HPRT*) were tested. The expression of *TBP*, *HPRT*, and *CHMP2A* mRNAs were identified as the least variable and, therefore, used as reference genes. The sequences are shown in the *Table* 1. Standard and melting curves were performed for every target to confirm primer efficiency and single product amplification, respectively. RT‐PCR data were analysed using the 2^(−ΔΔCT)^ method.^40^

**Table 1:** Reverse transcription polymerase chain reaction primer sequences used in Study 1

Target	Primer sequence
*TBP*, Forward	TGTGCTCACCCACCAACAAT
*TBP*, Reverse	TCTGCTCTGACTTTAGCACCTG
*CHMP2A*, Forward	CGCTATGTGCGCAAGTTTGT
*CHMP2A*, Reverse	GGGGCAACTTCAGCTGTCTG
*HPRT*, Forward	CCTGGCGTCGTGATTAGTGAT
*HPRT*, Reverse	TCGAGCAAGACGTTCAGTCC

### Study 2

#### Ethical approval

All animal procedures were conducted in accordance with institutional guidelines for the care and use of laboratory animals and were approved by the Institutional Animal Care and Use Committee of the University of Kentucky, which operates under the guidelines of the animal welfare act and the public health service policy on the humane care and use of laboratory animals. The study was conducted in adherence to the National Institutes of Health Guide for the Care and Use of Laboratory Animals.

#### Study design

Male Brown Norway/F344 rats at 10 months of age (National Institute on Aging, Bethesda, MD) were housed in a temperature and humidity controlled room and kept on a 12:12 h light : dark cycle with *ad libitum* access to food and water throughout the time course within the Division of Laboratory Animal Resources at the University of Kentucky. Rats were not fasted before euthanasia. Rats were randomly assigned to one of four groups: weight bearing conditions (WB; *n* = 5), HS for 24 h (HS 24 h; *n* = 4), HS for 7 days (HS 7d; *n* = 5), or HS for 7 days followed by 7 days of reloading (Rel; *n* = 5). The experimental design is depicted in *Figure* 1B. HS was performed as previously described.^36^ Briefly, a tail device containing a hook was attached with gauze and cyanoacrylate glue while the animals were anaesthetised with isoflurane (2% by inhalation). After the animal regained consciousness, the tail device was connected via a thin cable to a pulley sliding on a vertically adjustable stainless steel bar running longitudinally above a high sided cage. The system was designed in such a way that the rats could not rest their hind limbs against any side of the cage but could move about the cage on their front limbs and easily reach food and water. Rats in the Rel group were released from the tail suspension device after 7 days of unloading, and they were allowed to maintain normal ambulation for 7 days.

For determination of RNA synthesis and degradation rates, rats received a bolus of D_2_O (99%) prior to the start of the experiment, equivalent to 5% of the body water pool, followed by drinking water enriched 8% with D_2_O for the remainder of the experimental period. Specifically, HS rats received the bolus of D_2_O 2 days prior to the 7‐day suspension period. The WB rats were sacrificed 9 days after receiving the bolus and beginning D_2_O drinking water to match the labelling period of the HS group. Rats in the Rel group received the same bolus of D_2_O 2 days before the start of reambulation followed by 8% D_2_O‐enriched drinking water until euthanasia for a total of 9 days. These procedures are similar to previously published work.^36^, ^41^

At the end of the experimental period, between 8 a.m. and 12 p.m., rats were euthanized by an overdose of sodium pentobarbital via i.p. injection followed by exsanguination through a cardiac puncture. The plantaris and soleus muscles were dissected, weighed and frozen in liquid nitrogen before being stored at −80°C. Serum was isolated by allowing blood to clot at room temperature for 30 min before centrifugation for 10 min at 2000 *g* and 4°C. The serum supernatant was collected and stored at −80°C until analysis.

#### Total RNA extraction and reverse transcription polymerase chain reaction

Total RNA was extracted from ~25 mg of muscle tissue using TRI Reagent with the assistance of Direct‐zol kit (Zymo Research, Irvine, CA) following the manufacturer's instructions. Following cDNA synthesis using SuperScript IV VILO (Invitrogen, Carlsbad, CA), pre‐rRNA and mRNA of interest were measured by RT‐PCR. Quantitative PCR was performed using Fast SYBR Green master mix (Applied Biosystems, Foster City, CA) in a QuantStudio 3 real‐time PCR system (Thermo Fisher Scientific, Waltham, MA). Pre‐rRNAs were measured using primers designed specifically for a region spanning the 5.8S and second ITS of the 47S pre‐rRNA. The geometric mean of three reference genes^38^ was used for normalization. Several reference genes, among recently proposed stable genes,^39^ and genes usually found in the literature as reference genes (*Vcp*, *Tbp*, *Reep5*, *Hprt*, *Chmp2a*, *Gpi*, and *Emc7)* were tested. The expression of *Tbp*, *Reep5*, *Hprt*, and *Emc7* was identified as the least variable, and the geometric mean of these genes was used as reference. Standard and melting curves were performed for every target to confirm primer efficiency and single product amplification, respectively. Primer sequences used in Study 2 are shown in *Table* 2. Whole muscle total RNA content was calculated (total RNA concentration multiplied by the muscle weight) to estimate the total ribosomal mass in the entire muscle.

**Table 2:** Reverse transcription polymerase chain reaction primer sequences used in Study 2

Target	Primer sequence
*rpS6*, Forward	AGAGGAAGCGCAAGTCTGTC
*rpS6*, Reverse	CGACGAGGCACAGTGGTATC
*ITS1–5.8S*, Forward	ACACCTCAGATAACCGTACGACTC
*ITS1–5.8S*, Reverse	GCAATTCACATTAATTCTCGCAGC
*Tbp*, Forward	TGGGCTTCCCAGCTAAGTTC
*Tbp*, Reverse	CTGGCTCATAGCTACTGAACTGC
*Reep5*, Forward	TACTACATGCTGAAGTGCGGC
*Reep5*, Reverse	TGCTTCAGGAAGATGGGACG
*Hprt*, Forward	TACAGGCCAGACTTTGTTGG
*Hprt*, Reverse	TTGGCTTTTCCACTTTCGCTG
*Emc7*, Forward	CATGGGGCTGGACAGACTTC
*Emc7*, Reverse	CCGTCTCATGTCAGGGTCAC

#### RNA turnover

Total RNA extracted from Study 2 was used for ribosomal turnover analyses according to our published procedures.^36^, ^37^, ^42^, ^43^ In brief, the isolated RNA was hydrolysed overnight at 37°C with nuclease S1 and potato acid phosphatase. Hydrolysates were reacted with pentafluorobenzyl hydroxylamine and acetic acid and then acetylated with acetic anhydride and 1‐methylimidazole. Dichloromethane extracts were dried, resuspended in ethyl acetate and analysed on an Agilent 7890A GC coupled to an Agilent 5975C MS. For gas chromatography–mass spectrometry analysis, we used a DB‐17 column and negative chemical ionization, with helium as the carrier and methane as the reagent gas. The fractional molar isotope abundances at m/z 212 (M0) and 213 (M1) of the pentafluorobenzyl triacetyl derivative of purine ribose were quantified using MassHunter software. All analyses were corrected for abundance with an unenriched pentafluorobenzyl triacetyl purine ribose derivative standard.^36^, ^37^, ^42^, ^43^

Body water enrichment of D_2_O was performed using published procedures.^44^ Briefly, 120 μL of serum was placed into the inner well of O‐ring screw cap and inverted on an 80°C heating block for overnight distillation. Distilled samples were dilated 1:300 in ddH_2_O and analysed on a liquid water isotope analyser (Los Gatos Research, Los Gatos, CA, USA) against a standard curve prepared with 0–12% D_2_O.

The newly synthesized fraction of total RNA (~85% of total RNA exists as rRNA) was calculated by deuterium incorporation into purine ribose of RNA over the entire labelling period^36^, ^37^, ^42^, ^43^ with moderate iron deficiency anaemia adjustment of the equilibration of the enrichment of the body water pool with purine ribose.

#### Modelling calculations to account for non‐steady state conditions

We previously described disuse atrophy as a non‐steady state condition (i.e. rapid muscle loss), which violates an assumption of isotopic labelling.^36^ Ribosomal turnover (synthesis and degradation) was determined using previously published procedures for non‐steady state conditions.^36^, ^41^, ^45^ In brief, the mass of RNA at time *t*, *P*(*t*), obeys the differential equation:

(1) $$\frac{dP}{dt}=k_{\mathrm{syn}}-k_{\deg}P\left(t\right),$$

where *k*_*syn*_ is the synthesis rate, with dimensions of mass over time, and *k*_*deg*_ is the degradation constant, with dimensions of inverse time.

From equations derived in Miller et al.,^41^, ^45^ we calculate an equilibrium mass (*Peq*) and the rate parameters to reach that mass.

(2) $$k_{\mathrm{syn}}=k_{\deg}P_{\mathrm{eq}}.$$

#### Western blot

Following RNA extraction with TRI Reagent from Study 2, the organic phase was used for protein extraction. Following centrifugation, the protein pellet was solubilized using SDS‐urea buffer (100 mM Tris, pH 6.8, 12% glycerol, 4% SDS, 0.008% bromophenol blue, 2% *β*‐mercaptoethanol, 5 M urea) supplemented with Halt™ Protease (ThermoFisher #78438) and Phosphatase (ThermoFisher, #78426) Inhibitor Cocktails. Protein concentration was determined using RC DC™ Protein Assay (Bio‐Rad, Hercules, CA) with samples diluted to the same concentration with SDS‐urea buffer. Thirty micrograms of protein per sample was loaded on an SDS‐PAGE (10–18% gels, depending on the protein molecular weight of interest) and transferred to nitrocellulose membrane (Bio‐Rad). A pooled control sample was loaded in all gels for normalization. Membranes were blocked in 5% bovine serum albumin (#A‐420‐1, Gold Biotechnology, St. Louis, MO) for phospho‐specific antibodies or 5% non‐fat dry milk (#170‐6404, Bio‐Rad) for pan‐antibodies in Tris‐buffered saline with 0.1% Tween 20 (TBS‐T) for 2 h at room temperature. After blocking, membranes were incubated overnight at 4°C with one of the following primary antibodies (Cell Signaling Technology, Inc., Danvers, MA) in 5% bovine serum albumin in TBS‐T: phospho‐p70S6K ^Thr389^ (#9234, dilution 1:1000), total p70S6K (#9202, dilution 1:2000), ribosomal protein S6 (#2217, dilution 1:3000) and 4E‐BP1 (#9644, dilution 1:1000). After washing with TBS‐T, membranes were incubated with a goat anti‐rabbit (#G‐21234, Thermo Fisher Scientific) secondary antibody (dilution 1:10,000 in respective blocking solution) linked to horseradish peroxidase for 1 h at room temperature. Membranes were then incubated for 5 min with enhanced chemiluminescence reagent (Clarity Western ECL substrate, #170‐5060, Bio‐Rad) and exposed to a ChemiDoc™ MP Imaging System (Bio‐Rad). Bands were quantified using ImageJ software (NIH, Bethesda, MD). The hyperphosphorylated band (the top *γ* band) is presented as % of the total 4E‐BP1 bands.^46^ Phosphorylation of p70S6K ^thr389^ is normalized to total p70S6K. Total ribosomal protein S6 is normalized to Coomassie blue staining.

#### Statistical analysis

Data were analysed by one‐way repeated measures ANOVA (SigmaPlot v13.0, Systat Software, Inc., San Jose, CA, US) for Study 1, and one‐way ANOVA in Study 2. For both studies, Student–Newman–Keuls *post hoc* tests were used to determine the significance of pairwise comparisons. Normality was tested via Shapiro–Wilk test. When data were not normal distributed, data were log transformed prior to statistical analysis. Correlation analysis was performed using Pearson's *r* coefficient (two‐tailed). For Study 1, data are presented as whisker box representing interquartile ranges and the tails representing minimum to maximum values. Mean is indicated as cross and median as line. For Study 2, data are expressed as mean ± standard error of the mean in addition to the scatter dot plot for the individual points. Statistical significance was accepted at *P* ≤ 0.05.

## Results

### Limb immobilization in humans leads to decreased skeletal muscle size and is associated with reduced translational capacity

**FIGURE 2:**
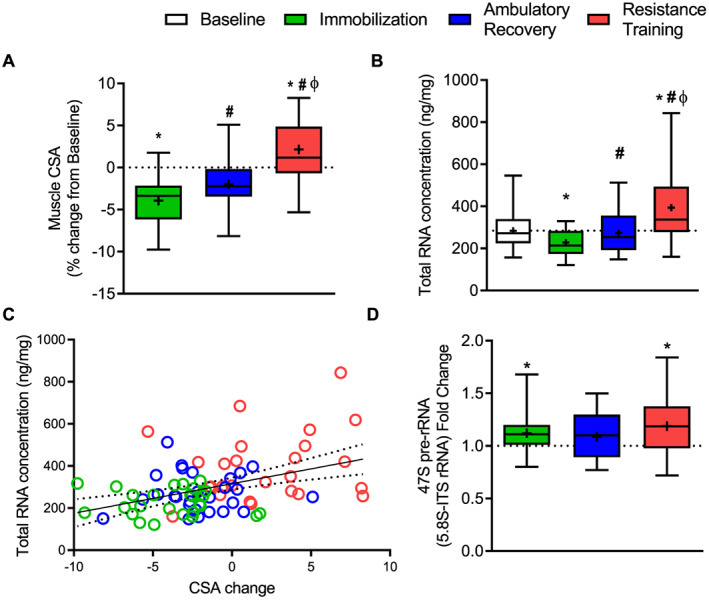
Effect of immobilization and recovery on translational capacity and ribosome biogenesis in humans. Percentage change of muscle CSA from baseline (*n* = 28) (A). Total RNA concentration (ng per mg of tissue weight) (B). Correlational analysis between changes in muscle size and changes in total RNA concentration (C). A 47S pre‐rRNA (5.8S‐ITS rRNA) fold change from baseline (D). Data are presented as box and whisker plots with the following representation: box (interquartile range), tails (minimum and maximum values), cross (mean), and line (median). Data in B and D failed normality test and were log‐transformed prior to statistical analysis. * indicates significantly different than baseline; #, significantly different than immobilization; ф, significantly different from ambulatory recovery (all *P* ≤ 0.05). CSA, cross‐sectional area.

In Study 1, 14 days of unilateral limb immobilization caused a ~4% reduction in muscle CSA in middle‐aged men (*Figure* 2A). Following 14 days of ambulatory recovery, muscle CSA was partially restored, and following a further 2 weeks of recovery combined with resistance training, it was significantly increased above baseline (*Figure* 2A). Limb immobilization for 14 days caused a 20% reduction in total RNA concentration (*Figure* 2B). This decrease was fully recovered to baseline levels with ambulatory recovery and increased further above baseline levels following 14 days of resistance training (*Figure* 2B). Muscle total RNA concentration (ng/mg) were positively correlated with changes in muscle CSA (*r* = 0.419, *P* < 0.001) (*Figure* 2C). To assess changes in rDNA transcription, we measured the long transcript of 47S pre‐rRNA via a primer set designed against the 5.8S rRNA spanning the ITS. Despite lower levels of total RNA concentration, there was a slight but significant increase in 47S pre‐rRNA level (~12%) after immobilization, while following ambulatory recovery, there was no difference from baseline (*Figure* 2D). Resistance training increased 47S pre‐rRNA above baseline levels by 19% (*Figure* 2D). The disconnection between total RNA concentration and 47S pre‐rRNA following limb immobilization in humans might be explained by the timing of the biopsy, which occurred 2 weeks after the start of muscle disuse. Because total RNA concentration was significantly down‐regulated at 14 days post immobilization, it is likely that rDNA transcription, as assessed by 47S pre‐rRNA, was down‐regulated earlier, which led us to investigate the acute effect of muscle disuse using the rodent model.

### Translational capacity is reduced with disuse and restored with reloading

**FIGURE 3:**
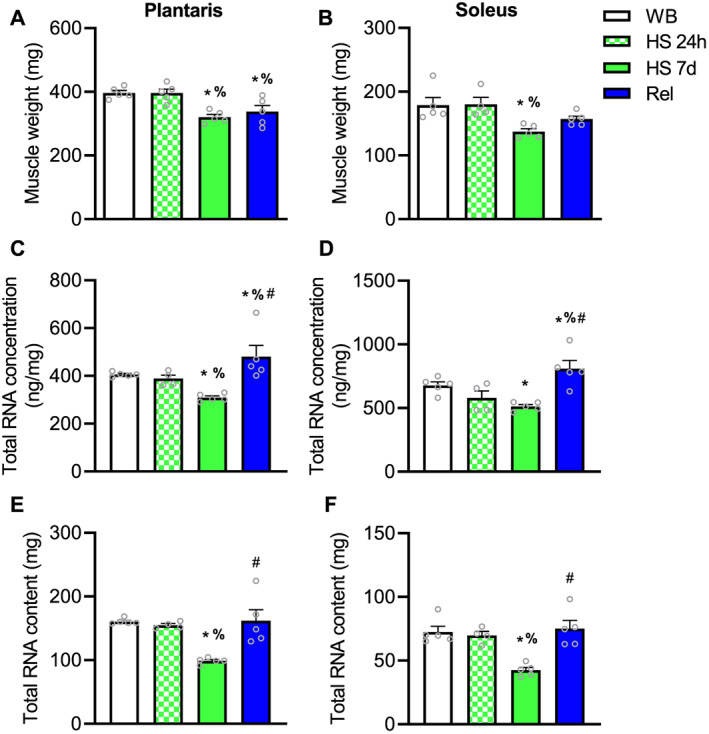
Effect of hindlimb suspension on muscle weight and translational capacity in rats. Plantaris muscle weight (A). Soleus muscle weight (B). Total RNA concentration (ng per mg of tissue) in plantaris muscle (C) and soleus (D). Total RNA content in plantaris muscle (E) and soleus muscle (F). Values are mean ± SEM (*n* = 4–5 per time point). Data in B, C, E, and F failed normality test and were log‐transformed prior to statistical analysis. * indicates significantly different than weight bearing; %, significantly different than HS 24 h; #, significantly different than HS 7d (all *P* ≤ 0.05). HS, hindlimb suspension; WB, weight bearing.

In Study 2, 7 days, but not 24 h, of HS (HS 7d) in rats resulted in substantial atrophy in both plantaris and soleus muscles (*Figure* 3A and 3B). Reloading (7 days of returning to normal ambulation) partially restored muscle mass in soleus, but not in plantaris muscle, which was still lower compared with WB (*Figure* 3A and 3B).

In both plantaris and soleus muscles, 24 h of HS did not result in differences in total RNA concentration or content when compared with WB (*Figure* 3C and 3D). However, translational capacity as measured by total RNA concentration and total RNA content was lower following 7 days of HS (*Figure* 3C–3F) in both soleus and plantaris muscles. Following 7 days of reloading, total RNA concentration was not only restored in both muscles but was also even higher than WB (*Figure* 3C and 3D), while total RNA content was restored to levels not different from WB (*Figure* 3E and 3F).

### Hindlimb suspension reduces ribosome biogenesis, while reloading restores it

**FIGURE 4:**
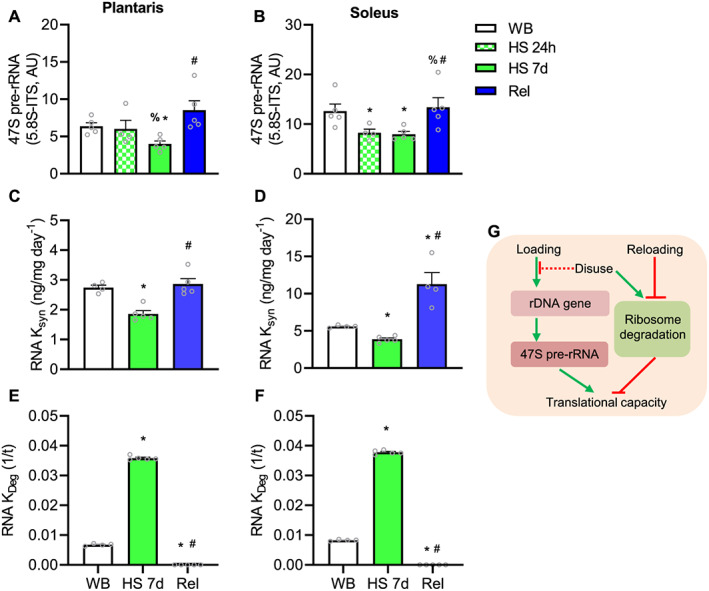
Effect of hindlimb suspension and reloading on ribosome biogenesis and ribosome degradation: 47S pre‐rRNA (5.8S‐ITS) in plantaris (A) and soleus (B). Rate of RNA synthesis (RNA *k*_*syn*_) in plantaris (C) and soleus (D). Rate of RNA degradation (*k*_*de*g_) in plantaris (E) and soleus (F). Values are mean ± SEM (*n* = 4–5 per time point). Data in A, B, D, and E failed normality test and were log‐transformed prior to statistical analysis. * indicates significantly different than weight bearing; %, significantly different than HS 24 h; #, significantly different than HS 7d (all *P* ≤ 0.05). Schematic representation of the effects of both loading and disuse on ribosome biogenesis and degradation affecting translational capacity (G). HS, hindlimb suspension; WB, weight bearing.

We hypothesized that if ribosome biogenesis is indeed important for muscle loss induced by disuse, decreased rDNA transcription should precede changes in translational capacity and muscle mass. In plantaris muscle, 47S pre‐rRNA was not impacted at 24 h of HS (*Figure* 4A). However, in soleus muscle, 47S pre‐rRNA was lower after 24 h of HS compared with WB (*Figure* 4B). Nevertheless, 47S pre‐rRNA was significantly lower in both plantaris and soleus at 7d HS (*Figure* 4A–4B). Reloading was associated with higher 47S pre‐rRNA compared with 7d HS in both plantaris and soleus muscle (*Figure* 4A and 4B) restoring it back to levels not different from WB. By using D_2_O to assess RNA turnover, we further confirmed the above observations by demonstrating that RNA synthesis (RNA K_syn_) was ~30% lower in 7 days HS compared with WB in both soleus and plantaris (*Figure* 4C and 4D). Furthermore, RNA *k*_syn_ in Rel was significantly higher compared with HS in plantaris restoring to WB levels, while in soleus, RNA *k*_syn_ was higher than HS as well as two‐fold higher than WB (*Figure* 4C and 4D). Synthesis of RNA is a measurement of ribosome biogenesis because most of the newly synthetized RNA is rRNA.^37^

### Ribosome degradation is markedly different with disuse and regrowth

Ribosome degradation (as measured by RNA *k*_*deg*_) was 5.3‐fold and 4.5‐fold higher in plantaris and soleus, respectively, after 7 days of HS (*Figure* 4E and 4F). After 7 days of reloading, ribosome degradation (RNA *k*_*deg*_) was significantly suppressed in both soleus and plantaris compared with HS and WB (*Figure* 4E–4F). The effects of HS on ribosome biogenesis and degradation are summarized on *Figure* 4G.

**FIGURE 5:**
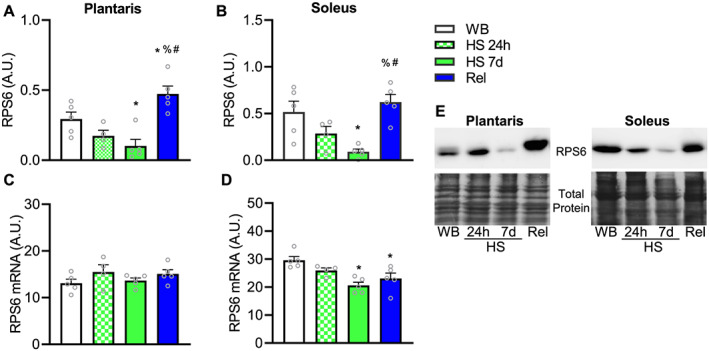
Effect of hindlimb suspension and reloading on ribosomal protein S6. Total levels of ribosomal protein S6 in plantaris (A) and soleus (B). mRNA levels of RPS6 in plantaris (C) and soleus (D). Values are mean ± SEM (*n* = 4–5 per time point). * indicates significantly different than weight bearing; %, significantly different than HS 24 h; #, significantly different than HS 7d (all *P* ≤ 0.05). Representative blots for RPS6 and total protein (Coomassie blue staining) are shown in E. HS, hindlimb suspension; mRNA, messenger RNA; WB, weight bearing.

To examine further the effect of unloading on ribosomal turnover, we measured protein levels of the ribosomal protein S6 (RPS6). RPS6 protein expression was not lower at 24 h after HS compared with WB in either the soleus or plantaris, but at 7 days, RPS6 protein levels was significantly lower in both muscles undergoing HS (*Figure* 5A–5B). After reloading, RPS6 protein levels were higher than WB and HS at both time points in plantaris (*Figure* 5B) but were similar to WB condition in soleus (*Figure* 5C). In contrast to protein levels, RPS6 mRNA levels were not different between groups in plantaris muscle (*Figure* 5C). However, in soleus, RPS6 mRNA was significantly lower in 7d HS and Rel compared with WB (*Figure* 5).

### Translational efficiency is affected during unloading and reloading

**FIGURE 6:**
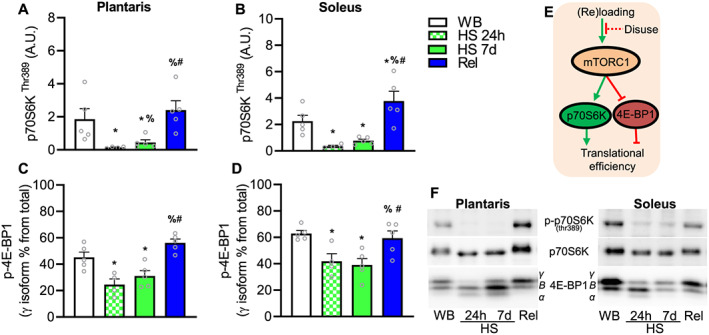
Effect of hindlimb suspension and reloading on mTORC1 signalling pathway. Phosphorylation of p70S6K at ^Thr389^, a direct target of mTORC1 in plantaris (A) and soleus (B). Measurement of the *γ* isoform (hyperphosphorylated state) of 4E‐BP1 in plantaris (C) and soleus (D). Values are mean ± SEM (*n* = 4–5 per time point). Data in B failed normality test and were log‐transformed prior to statistical analysis. * indicates significantly different than weight bearing; %, significantly different than HS 24 h; #, significantly different than HS 7d (all *P* ≤ 0.05). Representative blots and theoretical representation of the effect of loading and reloading on translational efficiency markers (J). HS, hindlimb suspension; WB, weight bearing.

We also investigated the effects of disuse and reloading on markers of translational efficiency, specifically the two bona‐fide mTORC1 direct targets, p70S6K ^Thr389^ and the phosphorylation state of 4E‐BP1. We assessed the phosphorylation status of 4E‐BP1 by the mobility shift of the different bands. Phosphorylation of both p70S6K ^Thr389,^ and the hyperphosphorylated *γ*‐band (top band) were significantly lower compared with WB condition at 24 h in both plantaris and soleus muscles (*Figure* 6A–6D). Both mTORC1 targets remained lower following 7 days of HS, in both muscles. Reloading restored mTORC1 activity in both soleus and plantaris muscles. Following 7 days, reloading re‐established phosphorylation of p70S6K ^Thr389^ and the hyperphosphorylated *γ*‐band to similar levels as WB condition (*Figure* 6A–6D). These cellular signalling events are summarized in *Figure* 6F.

## Discussion

The present study demonstrates that muscle disuse and reloading impact translation mechanisms at multiple levels. Muscle disuse reduced both markers of translational efficiency and capacity, specifically by suppressing mTORC1 signalling and ribosome biogenesis, respectively. Furthermore, ribosome degradation was substantially higher in muscle during disuse. Combined, decreased ribosome biogenesis and increased ribosome degradation lead to diminished translational capacity. In contrast, reloading restored translational efficiency and capacity by restoring mTORC1 activity and ribosome biogenesis while suppressing ribosome degradation.

Muscle atrophy due to disuse is associated with reduced protein synthesis in humans as well as rodents.^15^, ^16^, ^19^, ^20^ We hypothesized, compared with normal ambulation, disuse also lowers ribosome biogenesis resulting in lower translational capacity (total RNA concentration), helping explain lower protein synthesis rates observed during disuse. In the current study, 28 middle‐aged men underwent 2 weeks of unilateral limb immobilization, followed by 4 weeks of recovery (2 weeks of ambulatory recovery and 2 weeks of resistance training). Translational capacity was reduced in humans by ~20% in the unloaded *vastus lateralis* in the immobilized leg after 2 weeks. This finding is consistent with a previous study in five individuals with 10 days of muscle unloading that demonstrated a similar reduction (16%) in total RNA.^47^ However, in our study, 47S pre‐rRNA was not reduced post‐immobilization compared with baseline, indicating that we are unable to conclusively demonstrate that the decreased total RNA concentration was related to suppressed ribosome biogenesis. Considering the delayed time‐point of the muscle biopsy (14 days after immobilization), it is likely that we missed the early events that preceded the reduction in translational capacity, that is, lowered ribosome biogenesis. For this reason, we further investigated the early mechanistic events during muscle unloading in rats using the HS model. We focused our analyses at 24 h and 7 days post‐HS in two muscles of differing fibre‐type compositions and patterns of activation, namely, soleus and plantaris muscles. The soleus is a postural muscle high in type I, oxidative fibres while the plantaris is a mixed fibre type composition and functions during plantar flexion.^48^

Similar to the reduction of total RNA concentration after 14 days of immobilization in middle‐aged men, 7 days of HS in rats caused a reduction in muscle mass and a substantial decrease in translational capacity (total RNA concentration and content) in both soleus and plantaris. Importantly, 24 h of HS—while not a sufficient amount of time to observe a reduction in total RNA—caused a substantial decrease in 47S pre‐rRNA levels in rat soleus, although, 47S pre‐rRNA was not yet reduced at 24 h in plantaris muscle. Nevertheless, at 7 days, both muscles showed a reduction in 47S pre‐rRNA. By using D_2_O, which measures cumulative synthesis over the 7‐day period, we found that overall ribosome biogenesis (RNA synthesis) was reduced during the 7‐day period of muscle disuse in both muscles. The difference regarding the onset of reduced 47S pre‐rRNA expression between soleus and plantaris muscles suggests the activity pattern of a muscle influences the regulation of rDNA transcription. Soleus, as a postural muscle, may require minimal loading to maintain ribosome biogenesis compared with plantaris, indicated by the fact that rDNA transcription was lower immediately after the onset of disuse. Combined, these data suggest that normal ambulation, or a minimal load, is necessary to maintain basal rDNA transcription and that muscle disuse can rapidly decrease ribosome biogenesis, which remains suppressed throughout 7 days, resulting in reduced translational capacity following muscle disuse.

Muscle protein synthesis is rapidly decreased after the onset of muscle disuse in rodent models.^49^, ^50^, ^51^ Following several hours (~6 h), protein synthesis is down‐regulated, which is explained by reduced translational efficiency, as translational capacity (total RNA concentration) is not yet down‐regulated.^49^, ^50^ A lower rate of protein synthesis can be, at least partially, explained by down‐regulation of mTORC1 activity in the short‐term.^51^, ^52^ Indeed, we showed that the mTORC1 targets (p70S6K^thr389^ and phosphorylation status of 4E‐BP1) were rapidly hypophosphorylated at 24 h after HS and remained hypophosphorylated at 7 days. It is worth noting that rats drank and ate *ad libitum* until tissue collection, thus potentially impacting the interpretation of the findings herein, in particular, the signalling events related to mTORC1. However, the present findings that mTORC1 signalling is lower during disuse are consistent with previous studies.^51^, ^53^ As disuse progresses, translational capacity (total RNA concentration or total RNA content) also decreases.^50^, ^54^ Interestingly, Goldspink^50^ reported a decrease of 16% in total RNA concentration following 1 day of HS in rat soleus, similar to the 14% decrease we observed, although in our study the change failed to reach statistical significance (*P* = 0.14). In humans, sustained muscle disuse reduces protein synthesis both at rest^3^, ^4^ and in response to feeding.^20^ Furthermore, anabolic resistance to feeding may develop due to muscle disuse as a result of prolonged bed rest, hospitalization or overall reduced physical activity.^19^, ^55^ Using D_2_O, we previously showed that immobilization had little effect on the cumulative synthesis of myofibrillar protein during 14 days of human limb immobilization, although a significant effect on mitochondrial protein synthesis was observed.^6^ Thus, the data presented in this study may help explain the decreased protein synthesis rates in post‐prandial and post‐absorptive states and highlights that during muscle disuse, translational capacity is an important mediator of muscle protein synthesis rates.

While we and others have focused on ribosome biogenesis as a pivotal mechanism regulating muscle ribosomal mass, ribophagy (the specific lysosomal degradation of ribosomes) or other potential pathways degrading ribosomes may also play an important role in the maintenance of translational capacity.^30^, ^56^ Recently, we demonstrated that in gastrocnemius muscle of rats, RNA degradation (reflective primarily of rRNA) is higher during HS than normal loading.^36^ Consistent with our previous finding, in the current study, we show that compared with normal loading, RNA degradation was greater during disuse in both soleus and plantaris muscles, by 430% and 350%, respectively. Further, we found a robust reduction in ribosomal protein S6 (RPS6) content in both muscles at 7 days, which cannot be explained by its mRNA levels. Translation of the ribosomal protein mRNAs is under the control of mTORC1.^57^, ^58^ Thus, it is likely that synthesis of ribosomal proteins was down‐regulated during HS; however, the robust reduction in RPS6 is unlikely to be explained solely by reduced synthesis as ribosomal proteins have relative long half‐lives for non‐structural proteins. Ribosomal proteins in rat liver have a reported half‐life of 5–7 days on average.^37^, ^59^ Instead, the reduced levels of RPS6 corroborate the RNA degradations data that an active process, such as ribosome degradation, potentially via ribophagy, was a key mechanism reducing ribosomal mass during HS. The mechanism responsible for ribophagy remains elusive, but mTORC1 could be involved. In addition to its canonical role in translation initiation, mTORC1 has recently been shown to regulate ribophagy during starvation.^60^ Following mTORC1 inhibition, NUFIP1 binds to ribosomes targeting and delivering it to autophagosomes for degradation. Thus, it is possible that a repressed mTORC1 during HS helped to reduce rDNA transcriptional and also promoted ribophagy.

Muscle recovery after atrophy restores protein synthesis rates to levels that exceed that of normal WB conditions.^21^, ^43^ Returning to ambulation in rats increases both RNA synthesis and protein synthesis.^43^ We found markers of translational efficiency, such as the mTORC1 target p70S6K ^Thr389^ and the phosphorylation state of 4E‐BP1, were restored during reloading in both soleus and plantaris from rats. Moreover, the resumption of normal physical activity upon reloading restored translational capacity in both humans and rats. In our human investigation, 2 weeks of ambulatory recovery restored total RNA concentration. Furthermore, in our rodent study, 7 days of reloading following HS promoted ribosome biogenesis and suppressed ribophagy, restoring total RNA concentration. As stated above, mTORC1 is likely involved in regulating ribophagy as mTORC1 activity was rapidly restored during reloading, which coincided with suppressed ribosome degradation. Furthermore, RNA‐binding proteins (RBPs) play a key regulatory role in RNA stability and decay.^61^, ^62^ The suppression of degradation during reloading may be orchestrated by the stabilizing effects of RBPs on rRNA. RBPs may represent a critical node in controlling not only translation programming but also the translational capacity of muscle.^61^ As such, we have recently shown that RNA‐binding protein motif −3 induces hypertrophy and attenuates atrophy, which may be due to its role in RNA degradation.^63^ The high elevation in ribosome degradation with disuse and suppression during regrowth warrants future investigations. Clearly, more work in ribophagy and ribosome degradation and the potential regulation by mTORC1 and RBPs are necessary to better understand these mechanisms in skeletal muscle atrophy and regrowth.

Resistance training has been shown to promote muscle growth in conjunction with increased translational capacity.^26^, ^27^, ^28^ Similarly, in the current study, during the initial phase of muscle hypertrophy induced by resistance training (2 weeks), 47S pre‐rRNA was increased, and total RNA concentration was increased by 44% compared with post‐ambulatory recovery. These data provide further evidence showing that resistance training promotes ribosome biogenesis and increased translational capacity, which are important for muscle hypertrophy,^64^ in particular, as part of a recovery strategy following a muscle disuse episode.

Protein synthesis and ribosome biogenesis are the most energetic processes within most cell types.^65^, ^66^ Because of lack of muscle stimulation and reduced requirements to build or maintain muscle mass, it appears counterproductive to retain underused protein synthetic machinery. Maintenance of translational capacity may be costly, and thus, it is expected that ribosome biogenesis is down‐regulated during disuse. Ribosomes contain nucleosides (from rRNA) and amino acids (from ribosomal proteins) that can be utilized during starvation as a source of energy and nutrients via ribophagy, which is regulated by mTORC1.^60^ In our study, mTORC1 signalling was lower during HS, suggesting that under disuse conditions, the ribosomes may be degraded via a similar pathway to access its nucleoside and amino acid content. Reloading on the other hand not only promotes synthesis of new ribosomes but also reduces ribosome degradation. Ribosome degradation and biogenesis have been suggested to adjust to protein synthesis demands.^37^ In order to cope with growth requirements, our data suggest that muscle cells spare existing ribosomes for the increased demands of protein synthesis. In addition to ribosome biogenesis, this current study and the recent advances in the field from our groups indicate that ribosome degradation is a novel mechanism impacting on translational capacity during disuse.^36^ Also, the data presented in this study highlight for the first time that, in addition to increased ribosome biogenesis, suppression of ribosome degradation might be an important component increasing translational capacity during regrowth. While it seems tempting to judge the relative importance of RNA synthesis and degradation based on their respective magnitude of change, because RNA *k*_*syn*_ and *k*_*deg*_ are expressed in different units (ng/mg per day vs. 1/t, respectively), this comparison cannot be directly made.

**FIGURE 7:**
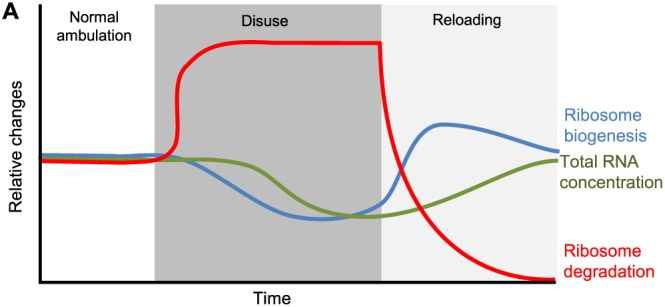
Theoretical model of contributors to translational capacity during muscle disuse and reloading. During normal ambulation or physical activity translational capacity is maintained via a balanced between ribosome biogenesis and degradation. After the onset of a muscle disuse event, ribosomal DNA transcription is inhibited, and degradation of ribosomes is increased, resulting in reduced translational capacity. Once normal ambulation is resumed, ribosome biogenesis is rapidly restored and ribosome degradation suppressed, restoring translational capacity.

The findings of the present study highlight the complexity and dynamic process induced by muscle disuse and reloading (summarized in *Figure* 7) and suggest that a minimal level of muscle activity is needed to maintain muscle ribosomal mass via ribosome biogenesis and degradation, therefore, contributing to muscle mass maintenance. One day of muscle disuse is sufficient to reduce mTORC1 signalling and rDNA transcription. Prolonged disuse decreases ribosome biogenesis and markedly increase ribosome degradation. The combination of down‐regulated rDNA transcription and up‐regulated ribophagy resulted in a reduced translational capacity and muscle loss. Thus, the decreases in both translational efficiency and capacity may help explain the lower rates of protein synthesis postabsorptive and in response to feeding previously observed during muscle disuse conditions.^3^, ^19^, ^67^ On the other hand, reloading results in muscle regrowth that is associated with restoration of mTORC1 activity and ribosome biogenesis together with robust suppression of ribosome degradation. In conclusion, the loss of muscle mass during disuse and subsequent regain in response to reloading are associated with changes in translational capacity. The present work demonstrates that maintenance of translational capacity via ribosome biogenesis and degradation could be a potential therapeutic target in the treatment of muscle atrophy resulting from disuse.

## Conflict of interest

All authors declare no conflicting financial interests.

## Funding

This work was funded by National Institute of Health//NCCIH grant AT009268 and in part by the New Zealand Primary Growth Partnership (PGP) post‐farm gate program, funded by Fonterra Co‐operative Group Ltd and the NZ Ministry for Primary Industries (MPI). The funders had no role in the data analysis or the production of the manuscript.

## References

[1] Arentson‐Lantz EJ , English KL , Paddon‐Jones D , Fry CS . Fourteen days of bed rest induces a decline in satellite cell content and robust atrophy of skeletal muscle fibers in middle‐aged adults. J Appl Physiol 2016;120:965–975. 26796754 10.1152/japplphysiol.00799.2015 PMC4835912

[2] Urso ML , Clarkson PM , Price TB . Immobilization effects in young and older adults. Eur J Appl Physiol 2006;96:564–571. 16369818 10.1007/s00421-005-0109-1

[3] Ferrando AA , Lane HW , Stuart CA , Davis‐Street J , Wolfe RR . Prolonged bed rest decreases skeletal muscle and whole body protein synthesis. Am J Physiol 1996;270:E627–E633. 8928769 10.1152/ajpendo.1996.270.4.E627

[4] Kortebein P , Ferrando A , Lombeida J , Wolfe R , Evans WJ . Effect of 10 days of bed rest on skeletal muscle in healthy older adults. JAMA 2007;297:1769. 10.1001/jama.297.16.1772-b 17456818

[5] Wall BT , Dirks ML , Snijders T , Senden JMG , Dolmans J , Van Loon LJC . Substantial skeletal muscle loss occurs during only 5 days of disuse. Acta Physiologica 2014;210:600–611. 24168489 10.1111/apha.12190

[6] Mitchell CJ , D'Souza RF , Mitchell SM , Figueiredo VC , Miller BF , Hamilton KL , et al. The impact of dairy protein during limb immobilization and recovery on muscle size and protein synthesis; a randomized controlled trial. J Appl Physiol 2018;124:717–728. 29122965 10.1152/japplphysiol.00803.2017 PMC6050201

[7] Kortebein P , Symons TB , Ferrando A , Paddon‐Jones D , Ronsen O , Protas E , et al. Functional impact of 10 days of bed rest in healthy older adults. J Gerontol A Biol Sci Med Sci 2008;63:1076–1081. 18948558 10.1093/gerona/63.10.1076

[8] Kouw IWK , Groen BBL , Smeets JSJ , Kramer IF , van Kranenburg JMX , Nilwik R , et al. One week of hospitalization following elective hip surgery induces substantial muscle atrophy in older patients. J Am Med Dir Assoc 2019;20:35–42. 30108034 10.1016/j.jamda.2018.06.018

[9] Wall BT , Dirks ML , van Loon LJ . Skeletal muscle atrophy during short‐term disuse: implications for age‐related sarcopenia. Ageing Res Rev 2013;12:898–906. 23948422 10.1016/j.arr.2013.07.003

[10] Chen L , Nelson DR , Zhao Y , Cui Z , Johnston JA . Relationship between muscle mass and muscle strength, and the impact of comorbidities: a population‐based, cross‐sectional study of older adults in the United States. BMC Geriatr 2013;13:74. 23865675 10.1186/1471-2318-13-74 PMC3765109

[11] Cherin P , Voronska E , Fraoucene N , De Jaeger C . Prevalence of sarcopenia among healthy ambulatory subjects: the sarcopenia begins from 45 years. Aging Clin Exp Res 2014;26:137–146. 24129803 10.1007/s40520-013-0132-8

[12] Janssen I , Heymsfield SB , Wang ZM , Ross R . Skeletal muscle mass and distribution in 468 men and women aged 18–88 yr. Journal of Applied Physiology (Bethesda, Md: 1985) 2000;89:81–88. 10.1152/jappl.2000.89.1.81 10904038

[13] English KL , Paddon‐Jones D . Protecting muscle mass and function in older adults during bed rest. Curr Opin Clin Nutr Metab Care 2010;13:34–39. 19898232 10.1097/MCO.0b013e328333aa66 PMC3276215

[14] Bell KE , von Allmen MT , Devries MC , Phillips SM . Muscle disuse as a pivotal problem in sarcopenia‐related muscle loss and dysfunction. J Frailty Aging 2016;5:33–41. 26980367 10.14283/jfa.2016.78

[15] Bodine SC . Disuse‐induced muscle wasting. International Journal of Biochemistry and Cell Biology 2013;45:2200–2208. 23800384 10.1016/j.biocel.2013.06.011 PMC3856924

[16] Smith HK , Matthews KG , Oldham JM , Jeanplong F , Falconer SJ , Bass JJ , et al. Translational signalling, atrogenic and myogenic gene expression during unloading and reloading of skeletal muscle in myostatin‐deficient mice. PloS one 2014;9:e94356‐e. 24718581 10.1371/journal.pone.0094356 PMC3981781

[17] Reid MB , Judge AR , Bodine SC . CrossTalk opposing view: the dominant mechanism causing disuse muscle atrophy is proteolysis. J Physiol 2014;592:5345–5347. 25512436 10.1113/jphysiol.2014.279406 PMC4270496

[18] Phillips SM , McGlory C . CrossTalk proposal: the dominant mechanism causing disuse muscle atrophy is decreased protein synthesis. J Physiol 2014;592:5341–5343. 25512435 10.1113/jphysiol.2014.273615 PMC4270495

[19] Wall BT , Dirks ML , Snijders T , van Dijk J‐W , Fritsch M , Verdijk LB , et al. Short‐term muscle disuse lowers myofibrillar protein synthesis rates and induces anabolic resistance to protein ingestion. Am J Physiol Endocrinol Metab 2016;310:E137–E147. 26578714 10.1152/ajpendo.00227.2015

[20] Glover EI , Phillips SM , Oates BR , Tang JE , Tarnopolsky MA , Selby A , et al. Immobilization induces anabolic resistance in human myofibrillar protein synthesis with low and high dose amino acid infusion. J Physiol 2008;586:6049–6061. 18955382 10.1113/jphysiol.2008.160333 PMC2655417

[21] Baehr LM , West DWD , Marcotte G , Marshall AG , De Sousa LG , Baar K , et al. Age‐related deficits in skeletal muscle recovery following disuse are associated with neuromuscular junction instability and ER stress, not impaired protein synthesis. Aging 2016;8:127–146. 26826670 10.18632/aging.100879 PMC4761718

[22] Heinemeier KM , Olesen JL , Haddad F , Schjerling P , Baldwin KM , Kjaer M . Effect of unloading followed by reloading on expression of collagen and related growth factors in rat tendon and muscle. J Appl Physiol 2009;106:178–186. 18988763 10.1152/japplphysiol.91092.2008

[23] Goldspink DF . The influence of activity on muscle size and protein turnover. J Physiol 1977;264:283–296. 839455 10.1113/jphysiol.1977.sp011668 PMC1307758

[24] Däpp C , Schmutz S , Hoppeler H , Flück M . Transcriptional reprogramming and ultrastructure during atrophy and recovery of mouse soleus muscle. Physiol Genomics 2004;20:97–107. 15479860 10.1152/physiolgenomics.00100.2004

[25] Lang SM , Kazi AA , Hong‐Brown L , Lang CH . Delayed recovery of skeletal muscle mass following hindlimb immobilization in mTOR heterozygous mice. PloS one 2012;7:e38910‐e. 22745686 10.1371/journal.pone.0038910 PMC3382153

[26] Figueiredo VC , Caldow MK , Massie V , Markworth JF , Cameron‐Smith D , Blazevich AJ . Ribosome biogenesis adaptation in resistance training‐induced human skeletal muscle hypertrophy. Am J Physiol Endocrinol Metab 2015;309:E72–E83. 25968575 10.1152/ajpendo.00050.2015

[27] Stec MJ , Kelly NA , Many GM , Windham ST , Tuggle SC , Bamman MM . Ribosome biogenesis may augment resistance training‐induced myofiber hypertrophy and is required for myotube growth in vitro. American Journal of Physiology Endocrinology and Metabolism 2016, ajpendo 00486 2015;310:E652–E661. 26860985 10.1152/ajpendo.00486.2015 PMC4835943

[28] Hammarstrom D , Ofsteng S , Koll L , Hanestadhaugen M , Hollan I , Apro W , et al. Benefits of higher resistance‐training volume are related to ribosome biogenesis. J Physiol 2020;598:543–565. 31813190 10.1113/JP278455

[29] Connolly M , Paul R , Farre‐Garros R , Natanek SA , Bloch S , Lee J , et al. miR‐424‐5p reduces ribosomal RNA and protein synthesis in muscle wasting. Journal of Cachexia Sarcopenia and Muscle 2018;9:400–416. 10.1002/jcsm.12266 PMC5879973 29215200

[30] Figueiredo VC , McCarthy JJ . Regulation of ribosome biogenesis in skeletal muscle hypertrophy. Physiology (Bethesda) 2019;34:30–42. 30540235 10.1152/physiol.00034.2018 PMC6383632

[31] Drygin D , Rice WG , Grummt I . The RNA polymerase I transcription machinery: an emerging target for the treatment of cancer. Annu Rev Pharmacol Toxicol 2010;50:131–156. 20055700 10.1146/annurev.pharmtox.010909.105844

[32] Cui C , Tseng H . Estimation of ribosomal RNA transcription rate in situ. Biotechniques 2004;36:134–138. 14740495 10.2144/04361RR04

[33] Luyken J , Hannan RD , Cheung JY , Rothblum LI . Regulation of rDNA transcription during endothelin‐1‐induced hypertrophy of neonatal cardiomyocytes. Hyperphosphorylation of upstream binding factor, an rDNA transcription factor. Circ Res 1996;78:354–361. 8593693 10.1161/01.res.78.3.354

[34] Panov KI , Friedrich JK , Zomerdijk JCBM . A step subsequent to preinitiation complex assembly at the ribosomal RNA gene promoter is rate limiting for human RNA polymerase I‐dependent transcription. Mol Cell Biol 2001;21:2641–2649. 11283244 10.1128/MCB.21.8.2641-2649.2001 PMC86895

[35] Popov A , Smirnov E , Kovacik L , Raska O , Hagen G , Stixova L , et al. Duration of the first steps of the human rRNA processing. Nucleus 2013;4:134–141. 23412654 10.4161/nucl.23985 PMC3621745

[36] Lawrence MM , Van Pelt DW , Confides AL , Hunt ER , Hettinger ZR , Laurin JL , et al. Massage as a mechanotherapy promotes skeletal muscle protein and ribosomal turnover but does not mitigate muscle atrophy during disuse in adult rats. Acta Physiol (Oxf) 2020;229:e13460. 32125770 10.1111/apha.13460 PMC7293583

[37] Mathis AD , Naylor BC , Carson RH , Evans E , Harwell J , Knecht J , et al. Mechanisms of in vivo ribosome maintenance change in response to nutrient signals. Mol Cell Proteomics 2017;16:243–254. 27932527 10.1074/mcp.M116.063255 PMC5294211

[38] Vandesompele J , De Preter K , Pattyn F , Poppe B , Van Roy N , De Paepe A , et al. Accurate normalization of real‐time quantitative RT‐PCR data by geometric averaging of multiple internal control genes. Genome Biology 2002;3: RESEARCH0034‐RESEARCH. 10.1186/gb-2002-3-7-research0034 PMC126239 12184808

[39] Eisenberg E , Levanon EY . Human housekeeping genes, revisited. Trends in genetics: TIG 2013;29:569–574. 23810203 10.1016/j.tig.2013.05.010

[40] Livak KJ , Schmittgen TD . Analysis of relative gene expression data using real‐time quantitative PCR and the 2−ΔΔCT method. Methods 2001;25:402–408. 11846609 10.1006/meth.2001.1262

[41] Miller BF , Hamilton KL , Majeed ZR , Abshire SM , Confides AL , Hayek AM , et al. Enhanced skeletal muscle regrowth and remodelling in massaged and contralateral non‐massaged hindlimb. J Physiol 2018;596:83–103. 29090454 10.1113/JP275089 PMC5746529

[42] Sieljacks P , Wang J , Groennebaek T , Rindom E , Jakobsgaard JE , Herskind J , et al. Six weeks of low‐load blood flow restricted and high‐load resistance exercise training produce similar increases in cumulative myofibrillar protein synthesis and ribosomal biogenesis in healthy males. Front Physiol 2019;10:649. 31191347 10.3389/fphys.2019.00649 PMC6548815

[43] Miller BF , Baehr LM , Musci RV , Reid JJ , Peelor FF 3rd , Hamilton KL , et al. Muscle‐specific changes in protein synthesis with aging and reloading after disuse atrophy. J Cachexia Sarcopenia Muscle 2019;10:1195–1209. 31313502 10.1002/jcsm.12470 PMC6903438

[44] Groennebaek T , Sieljacks P , Nielsen R , Pryds K , Jespersen NR , Wang J , et al. Effect of blood flow restricted resistance exercise and remote ischemic conditioning on functional capacity and myocellular adaptations in patients with heart failure. Circ Heart Fail 2019;12:e006427. 31830830 10.1161/CIRCHEARTFAILURE.119.006427

[45] Miller BF , Wolff CA , Peelor FF 3rd , Shipman PD , Hamilton KL . Modeling the contribution of individual proteins to mixed skeletal muscle protein synthetic rates over increasing periods of label incorporation. J Appl Physiol 2015;118:655–661. 25593288 10.1152/japplphysiol.00987.2014 PMC4360018

[46] Anthony JC , Reiter AK , Anthony TG , Crozier SJ , Lang CH , MacLean DA , et al. Orally administered leucine enhances protein synthesis in skeletal muscle of diabetic rats in the absence of increases in 4E‐BP1 or S6K1 phosphorylation. Diabetes 2002;51:928–936. 11916909 10.2337/diabetes.51.4.928

[47] Gamrin L , Berg HE , Essén P , Tesch PA , Hultman E , Garlick PJ , et al. The effect of unloading on protein synthesis in human skeletal muscle. Acta Physiol Scand 1998;163:369–377. 9789580 10.1046/j.1365-201X.1998.t01-1-00391.x

[48] Bloemberg D , Quadrilatero J . Rapid determination of myosin heavy chain expression in rat, mouse, and human skeletal muscle using multicolor immunofluorescence analysis. PloS one 2012;7:e35273. 22530000 10.1371/journal.pone.0035273 PMC3329435

[49] Booth FW , Seider MJ . Early change in skeletal muscle protein synthesis after limb immobilization of rats. J Appl Physiol 1979;47:974–977. 511723 10.1152/jappl.1979.47.5.974

[50] Goldspink DF . The influence of immobilization and stretch on protein turnover of rat skeletal muscle. J Physiol 1977;264:267–282. 839454 10.1113/jphysiol.1977.sp011667 PMC1307757

[51] Kelleher AR , Kimball SR , Dennis MD , Schilder RJ , Jefferson LS . The mTORC1 signaling repressors REDD1/2 are rapidly induced and activation of p70S6K1 by leucine is defective in skeletal muscle of an immobilized rat hindlimb. Am J Physiol Endocrinol Metab 2013;304:E229–E236. 23193052 10.1152/ajpendo.00409.2012 PMC3543567

[52] Hornberger TA , Hunter RB , Kandarian SC , Esser KA . Regulation of translation factors during hindlimb unloading and denervation of skeletal muscle in rats. Am J Physiol Cell Physiol 2001;281:C179–C187. 11401840 10.1152/ajpcell.2001.281.1.C179

[53] Roberson PA , Shimkus KL , Welles JE , Xu D , Whitsell AL , Kimball EM , et al. A time course for markers of protein synthesis and degradation with hindlimb unloading and the accompanying anabolic resistance to refeeding. J Appl Physiol 2020;129:36–46. 32407240 10.1152/japplphysiol.00155.2020 PMC7469230

[54] Mirzoev T , Tyganov S , Vilchinskaya N , Lomonosova Y , Shenkman B . Key markers of mTORC1‐dependent and mTORC1‐independent signaling pathways regulating protein synthesis in rat soleus muscle during early stages of hindlimb unloading. Cell Physiol Biochem 2016;39:1011–1020. 27536969 10.1159/000447808

[55] Burd NA , Gorissen SH , Van Loon LJC . Anabolic resistance of muscle protein synthesis with aging. Exerc Sport Sci Rev 2013;41:169–173. 23558692 10.1097/JES.0b013e318292f3d5

[56] Kraft C , Deplazes A , Sohrmann M , Peter M . Mature ribosomes are selectively degraded upon starvation by an autophagy pathway requiring the Ubp3p/Bre5p ubiquitin protease. Nat Cell Biol 2008;10:602–610. 18391941 10.1038/ncb1723

[57] Goodman CA . Role of mTORC1 in mechanically induced increases in translation and skeletal muscle mass. J Appl Physiol 2019;127:581–590. 30676865 10.1152/japplphysiol.01011.2018

[58] Thoreen CC , Chantranupong L , Keys HR , Wang T , Gray NS , Sabatini DM . A unifying model for mTORC1‐mediated regulation of mRNA translation. Nature 2012;485:109–113. 22552098 10.1038/nature11083 PMC3347774

[59] Hirsch CA , Hiatt HH . Turnover of liver ribosomes in fed and in fasted rats. J Biol Chem 1966;241:5936–5940. 5954370

[60] Wyant GA , Abu‐Remaileh M , Frenkel EM , Laqtom NN , Dharamdasani V , Lewis CA , et al. NUFIP1 is a ribosome receptor for starvation‐induced ribophagy. Science 2018;360:751–758. 29700228 10.1126/science.aar2663 PMC6020066

[61] Van Pelt DW , Hettinger ZR , Vanderklish PW . RNA‐binding proteins: the next step in translating skeletal muscle adaptations? J Appl Physiol 2019;127:654–660. 31120811 10.1152/japplphysiol.00076.2019

[62] Kaiser RWJ , Ignarski M , Van Nostrand EL , Frese CK , Jain M , Cukoski S , et al. A protein‐RNA interaction atlas of the ribosome biogenesis factor AATF. Sci Rep 2019;9:11071. 31363146 10.1038/s41598-019-47552-3 PMC6667500

[63] Van Pelt DW , Confides AL , Judge AR , Vanderklish PW , Dupont‐Versteegden EE . Cold shock protein RBM3 attenuates atrophy and induces hypertrophy in skeletal muscle. J Muscle Res Cell Motil 2018;39:35–40. 30051360 10.1007/s10974-018-9496-x

[64] Figueiredo VC . Revisiting the roles of protein synthesis during skeletal muscle hypertrophy induced by exercise. Am J Physiol Regul Integr Comp Physiol 2019;317:R709–R718. 31508978 10.1152/ajpregu.00162.2019

[65] Buttgereit F , Brand MD . A hierarchy of ATP‐consuming processes in mammalian cells. Biochem J 1995;312:163–167. 7492307 10.1042/bj3120163 PMC1136240

[66] Warner JR . The economics of ribosome biosynthesis in yeast. Trends Biochem Sci 1999;24:437–440. 10542411 10.1016/s0968-0004(99)01460-7

[67] Kilroe SP , Fulford J , Jackman S , Holwerda A , Gijsen A , van Loon L , et al. Dietary protein intake does not modulate daily myofibrillar protein synthesis rates or loss of muscle mass and function during short‐term immobilization in young men: a randomized controlled trial. Am J Clin Nutr 2020; 10.1093/ajcn/nqaa136 32469388

[68] von Haehling S , Morley JE , Coats AJS , Anker SD . Ethical guidelines for publishing in the Journal of Cachexia, Sarcopenia and Muscle: update 2019. J Cachexia Sarcopenia Muscle 2019;10:1143–1083. 31661195 10.1002/jcsm.12501 PMC6818444

